# Virus-specific memory T cells populate tumors and can be repurposed for tumor immunotherapy

**DOI:** 10.1038/s41467-019-08534-1

**Published:** 2019-02-04

**Authors:** Pamela C. Rosato, Sathi Wijeyesinghe, J. Michael Stolley, Christine E. Nelson, Rachel L. Davis, Luke S. Manlove, Christopher A. Pennell, Bruce R. Blazar, Clark C. Chen, Melissa A. Geller, Vaiva Vezys, David Masopust

**Affiliations:** 1 Department of Microbiology and Immunology, Center for Immunology, University of Minnesota, Minneapolis, MN 55455 USA; 2 Department of Laboratory Medicine and Pathology, Center for Immunology, University of Minnesota, Minneapolis, MN 55455 USA; 3 Department of Pediatrics, Center for Immunology, University of Minnesota, Minneapolis, MN 55455 USA; 4 Department of Neurosurgery, University of Minnesota, Minneapolis, MN 55455 USA; 5 Department of Obstetrics, Gynecology and Women’s Health, University of Minnesota, Minneapolis, MN 55455 USA

## Abstract

The immunosuppressive tumor microenvironment limits the success of current immunotherapies. The host retains memory T cells specific for previous infections throughout the entire body that are capable of executing potent and immediate immunostimulatory functions. Here we show that virus-specific memory T cells extend their surveillance to mouse and human tumors. Reactivating these antiviral T cells can arrest growth of checkpoint blockade-resistant and poorly immunogenic tumors in mice after injecting adjuvant-free non-replicating viral peptides into tumors. Peptide mimics a viral reinfection event to memory CD8+ T cells, triggering antigen presentation and cytotoxic pathways within the tumor, activating dendritic cells and natural killer cells, and recruiting the adaptive immune system. Viral peptide treatment of ex vivo human tumors recapitulates immune activation gene expression profiles observed in mice. Lastly, peptide therapy renders resistant mouse tumors susceptible to PD-L1 blockade. Thus, re-stimulating known antiviral immunity may provide a unique therapeutic approach for cancer immunotherapy.

## Abstract (web summary)

The immunosuppressive tumor environment and the lack of functional anti-tumor immunity are major limiting factors in immunotherapy. Here the authors show that human and mouse tumors are infiltrated by virus-specific memory T cells, which can be harnessed by viral peptides to induce local and systemic anti-tumor immunity and synergize with checkpoint blockade.

## Introduction

Tumor immunotherapy has revolutionized cancer treatment. Current therapies, however, remain suboptimal and are often not effective in many patients. Therapies must contend with exhaustion of tumor-specific T cells, and checkpoint blockade therapies aimed at reversing this exhaustion are only efficacious in a subset of patients^1^. Vaccines that carry tumor antigens are also aimed at reinvigorating exhausted cells or priming new responses, however, these therapies suffer from the need to identify rare immunogenic epitopes that are often personalized (patient-specific) and subject to tumor escape. Adoptive cell therapies (ACT) bypass tumor-specific T-cell exhaustion issues and have been largely successful at eliminating blood cancers. Application to solid tumors, however, has rarely been effective, and in addition to epitope identification, T-cell migration to the tumor is a major barrier^2^. All therapies must also contend with the immunosuppressive tumor microenvironment^3^. Strategies to do so include intratumoral injection of live oncolytic virus, which can kill tumor cells and promote an inflammatory antiviral response, however, this property can be self-limiting by inducing an antiviral response that then inhibits the viral therapy. Other strategies include intratumoral injection of microbial products that target an innate immune signaling pathway, such as toll-like receptors or STING^4^. It is clear current immunotherapy approaches have great promise for subsets of patients, however, new therapeutic approaches are needed.

Humans experience many viral infections. Once controlled, the host retains memory CD8 + T cells throughout the entire body to sense reinfection or recrudescence^5,6^. These antiviral memory T cells are licensed to respond quickly, remain highly vigilant, are capable of cytotoxicity, and are plentiful throughout the body. In addition to killing targeted cells, when antiviral memory CD8 + T cells encounter the specific peptides that they were primed to respond to during the initial infection, they interpret this rapid recognition as a reinfection event and promote potent local immunoactivation, locally orchestrating immune defenses at that site. This property is no longer strictly dependent on innate signals needed for a primary T-cell response, and it occurs within mere hours of peptide exposure^7–9^. Unlike human tumor antigens, which can be patient-specific and non-immunogenic, the immunogenic peptides recognized by virus-specific CD8 + T cells are widely known for common human pathogens. We asked whether antiviral memory CD8 + T cells could be triggered by peptides for cancer immunotherapy.

Here we observe that, like healthy tissue, mouse and human tumors are commonly surveyed by memory T cells specific for previously encountered viral infections, and these functional T cells can be specifically reactivated via local delivery of adjuvant-free viral peptide. Antiviral T-cell reactivation induces activation of both the innate and adaptive immune system within the tumor, arrests tumor growth, and synergizes with PD-L1 checkpoint blockade to eliminate normally resistant tumors. Immune activation was observed in human tumors treated ex vivo with viral-derived peptides, supporting that natural and existing antiviral immunity is abundant in solid tumors and can be repurposed as a tumor immunotherapy.

## Results

### Antiviral memory T-cell activation arrests tumor growth

**Fig. 1:**
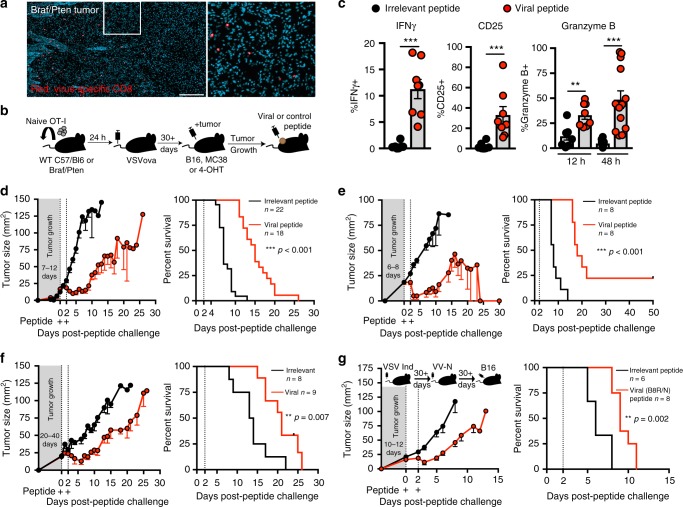
Antiviral memory T-cell activation arrests tumor growth. **a** Immunofluorescence staining of *Braf/Pten* tumor (Red, OT-I; teal, 4′,6-diamidino-2-phenylindole (DAPI)-stained nuclei. Scale bar = 250 μm. **b** Schematic of experimental set up. **c** Proportion of IFNγ +, CD25 + and granzyme B + OT-I in B16 melanoma tumors 12 h following intratumoral irrelevant (black circles) or viral SIINFEKL peptide (red circles) with *n* = 8 mice (12 h), and *n* = 13 mice (48 h). **d**–**g** Tumor growth (left) and survival (right) following two intratumoral peptide injections 48h apart in OT-I chimeras with B16 melanoma (**d**), OT-I chimeras with MC38 (**e**), *Braf/Pten* OT-I chimeras (**f**), or mice with endogenous memory generated to VSV Indiana and VV-N (**g**). Black lines denote irrelevant control peptide and red lines denote viral peptide in figures (**d**–**g**). Sample size for tumor growth and survival plots indicated in the figure where *n* = number of mice. Significance was determined by unpaired two-tailed Mann–Whitney (IFNγ and CD25), and unpaired two-tailed *t*-test (granzyme B) for (**c**), and Log-rank Mantel–Cox test for (**d**–**g**) where **p < 0.01, ***p < 0.001. **a** Image representative of three tumors. **c**, **d**, **f**, Data pooled from three independent experiments. **e**, **g**, Data pooled from two independent experiments. Lines represent means and error bars are SEM

To visualize whether mouse tumors were surveyed by memory T cells specific for acute viral infections, we established mouse models that contained antiviral CD8 + T cells bearing markers compatible with immunohistochemistry, which favored the use of an antiviral transgenic T-cell population bearing a stainable marker, CD45.1. CD45.1 + OT-I transgenic OVA peptide-specific CD8 + T cells were transferred to naive mice. The following day, recipients were infected with live replicating vesicular stomatitis virus expressing OVA (VSVova), which resulted in the establishment of broadly distributed OT-I memory CD8 + T cells (Fig. 1a). These mice are referred to as OT-I chimeras. To test whether developing tumors would be populated by pre-existing memory CD8 + T cells, OT-I chimeras were inoculated with 1.5 × 10^5^ aggressively growing B16 melanoma cells i.d. Alternatively, OT-I chimeras were generated in *BRaf*^*CA*^*,Pten*^*loxP*^*,Tyr::Cre-ER*^*T2*^ mice (herein referred to as *Braf/Pten*), which develop local autochthonous tumors in skin after topical application of tamoxifen through Cre-mediated deletion of the tumor suppressor *Pten* and expression of the mutant *Braf*^*V600E*^ oncogene in melanocytes^10^. In both cases, previously established antiviral memory T cells extended immunosurveillance to tumors, consistent with previous reports^11^ (Fig. 1a and Supplementary Figure 1).

We next asked if antiviral T cells within the tumor microenvironment were amenable to peptide-mediated reactivation, as we first observed in healthy skin (Supplementary Figure 2). OVA peptide, without adjuvant, was injected into palpable tumors (Fig. 1b). This peptide is a synthetic mimic of the viral epitope that originally induced the OT-I response, but importantly, is not expressed by the tumor. Within 12 h, intratumoral antiviral CD8+ T cells expressed IFNγ, CD25, and cytotoxic granyzme B (Fig. 1c), indicating that antiviral CD8 + T cells within tumors become activated in response to local viral peptide injection.

We observed significant delays in tumor growth kinetics in response to antiviral T-cell reactivation (Fig. 1d–f and Supplementary Figure 3). This was true when viral peptide was injected into palpable, poorly immunogenic melanoma tumors of *Braf/Pten* mice, or into MC38 colon adenocarcinoma or B16 melanoma i.d. injected tumor cell lines, with total clearance of the moderately immunogenic MC38 observed in some mice (Fig. 1d–f and Supplementary Figure 3). GP33 peptide from a different virus (LCMV) that had not been experienced by these mice had no effect, indicating that tumor control was dependent on antigen-specific reactivation of pre-existing antiviral immunity. We also tested growth of B16 tumors in mice that did not receive OT-I T cells but rather had endogenous B8R and N-specific memory CD8+ T cells due to prior exposure to vesicular stomatitis virus (VSV) and recombinant vaccinia virus expressing the N epitope derived from VSV; tumor control was similar in response to N and B8R peptides, indicating that this phenomenon did not depend on the experimental use of transgenic T cells (Fig. 1g).

### Viral peptides promote intratumoral immune activation

**Fig. 2:**
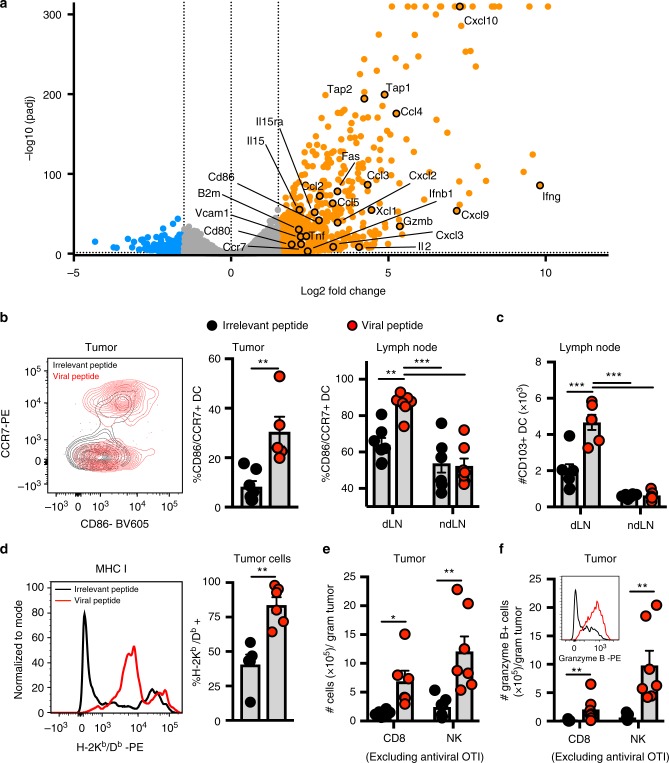
Antiviral T-cell reactivation promotes intratumoral immune activation. **a** Volcano plot of differentially expressed genes determined by RNAseq from whole B16 tumors 9 h after peptide exposure. Orange and blue circles are significantly (*q*-value < 0.05) upregulated (>1.5 log_2_FC) or downregulated genes (<−1.5 log_2_FC), respectively, compared to irrelevant peptide treated tumors (*n* = 3 mice; data from one experiment). **b** Proportion of CD86 +/CCR7 + CD103+ DCs in tumor at 12 h (*n* = 6 mice for irrelevant, *n* = 5 mice for viral), and tumor draining (dLN) or non-draining (ndLN) LN at 48 h (*n* = 7) following intratumoral irrelevant (black circles) or viral SIINFEKL peptide (red circles). **c** Quantification of CD103 + DCs in LN at 48 h. *n* = 6 mice (irrelevant), *n* = 7 mice (viral). **d** Proportion of B16 tumor cells expressing MHC I at 24 h. *n* = 5 mice (irrelevant), *n* = 6 mice (viral). **e** Quantification of NK cells and CD44hi CD8+ T cells (excluding OT-I) in *Braf/Pten* tumors (48 h). *n* = 6 mice (CD8), and *n* = 7 mice (NK). **f** Enumeration of granzyme B + NK and non-viral peptide-specific CD8+ T cells in *Braf/Pten* tumors. Example of granzyme B staining in NK cells (inset) at 48 h; *n* = 7 mice. All data, unless indicated, are pooled from at least two independent experiments. Significance was determined by unpaired two-tailed Mann–Whitney test (**e**, CD8); and unpaired two-tailed *t*-test (**b**, tumor) **d**, and (**e**, NK), and a one-way unpaired ANOVA with Tukey post hoc test (**b**, **LN**) and (**c**). Lines represent means and error bars are SEM. *p < 0.05, **p < 0.01, ***p < 0.001

To explore potential mechanisms, we profiled B16 tumors 9 h after peptide therapy by RNAseq. Many genes associated with immune activation were significantly upregulated (Fig. 2a). This included CD80, CD86, and CCR7, all associated with dendritic cell (DC) activation and migration to draining lymph nodes. As CD103 + DCs have been described as critical for priming anti-melanoma T-cell responses^12^, we tested if this subset was activated after therapy. We observed that CD103 + dendritic cells had indeed upregulated CD86 and CCR7 within the tumor after 12 h (Fig. 2b), and observed a corresponding increase in activation and accumulation within the draining lymph node at 48 h (Fig. 2b, c). Tap1, Tap2, and β2M genes were also upregulated, which are involved in natural killer cell recognition, and tumor cell processing of antigens and presentation through MHC I to cytotoxic CD8+ T cells. Indeed, we found that tumor cells notably increased MHC I expression within 24 h of peptide therapy (Fig. 2d). This finding, coupled with the observed rapidity of tumor control (Fig. 1d–g) and upregulation of the tumoricidal molecule granzyme B (Fig. 1c), suggests direct killing of peptide-coated tumor cells by reactivated antiviral T cells may have occurred, as has been previously described for vaccine-generated T cells^13^. Chemokines and VCAM-1, used to recruit immune cells, were also expressed and this was accompanied by an accumulation of intratumoral CD8+ T cells (excluding antiviral OT-I) and natural killer cells, both of which upregulated granzyme B (Fig. 2e, f). Fas, also a mechanism by which tumor cells are killed by CD8+ T cells and NK cells, was also upregulated in response to peptide therapy (Fig. 2a). Taken together, peptide therapy promotes a broad immune-activating environment, promoting the accumulation of CD8+ T cells, NK cells, and DCs within tumors, activating DCs within draining LNs, increasing MHC I expression by tumor cells, and upregulating cytotoxic molecules by cells associated with tumor control.

### Viral peptides promote immune activation in human tumors

**Fig. 3:**
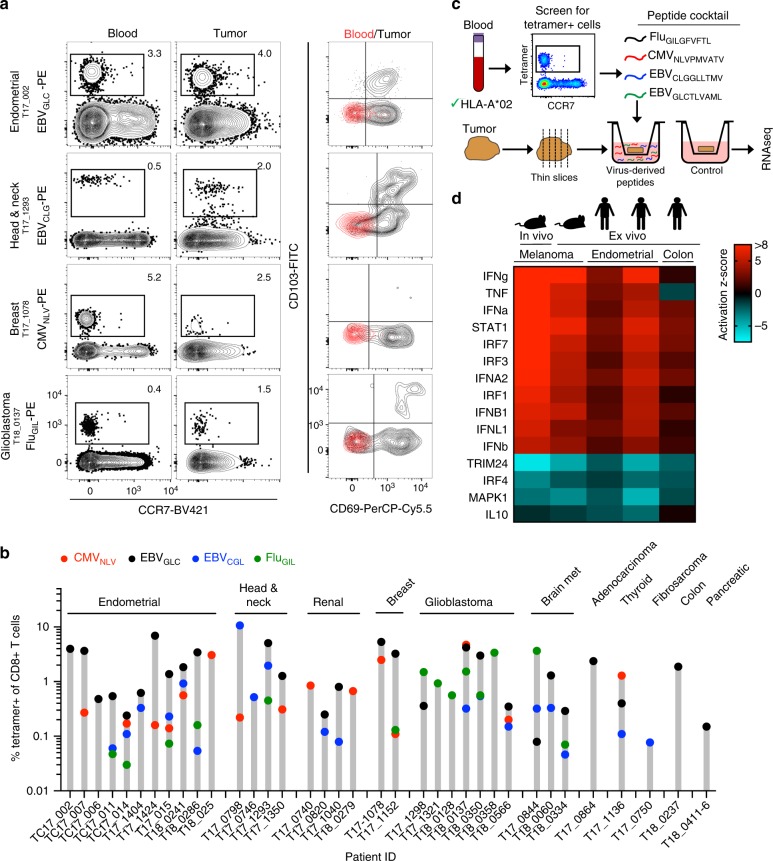
Antiviral T cells populate human tumors and can promote immune activation. **a** Flow plots of human tumors and paired blood stained for HLA-A*02 + tetramers specific for EBV (EBV_GLC_ and EBV_CLG_), CMV (CMV_NLV_), and Influenza (Flu_GIL_) (showing 4 of 36 patients, as indicated (**b**). Left two columns gated on CD8 +/CD3 + cells. Right column; CD69 and CD103 phenotype of tetramer-positive cells. **b** Frequency of tetramer + T cells in all human tumors analyzed. Each bar is a patient. Symbol color represents specific tetramer; red circles = CMV_NLV_, black circles = EBV_GLC_, blue circles = EBV_CGL_, green circles = Flu_GIL_. **c** Schematic of human organotypic slice culture experimental design. **d** Upstream transcriptional regulator analysis using ingenuity pathway analysis (IPA) software on differentially expressed genes after 9 h treatment with control or viral peptide with a *q*-value < 0.1 from in vivo mouse B16 tumors (*n* = 3 mice), ex vivo *Braf/Pten* tumors slice culture (*n* = 3 mice) or two distinct human endometrial (from three technical replicates each) or one colon (from two technical replicates) tumor slice cultures (colors denote activation *z*-score). Patient IDs in order from left to right are T18_0241, T17_1424, and T18_0237. Upstream transcriptional regulators could not be identified by IPA in a fourth human tumor sample (T18_0286), as no significantly differentially expressed genes were identified (see Supplementary Table 2)

We next assessed whether antiviral CD8+ T cell immunosurveillance commonly extended to multiple human tumor types. Because ~50% of Minnesotans express HLA-A*02, we generated HLA-A*02 MHC I tetramers containing known immunogenic peptides from the common viral infections Epstein–Barr virus (EBV), cytomegalovirus (CMV), and influenza (Flu). In cases where paired blood samples were obtainable (> 90%) and sufficient T cells could be isolated from available tumor sample for analysis (77%), identification of virus-specific T cells in the blood was 100% predictive of the presence of the same population in tumors, including brain metastases, glioblastoma, renal cell carcinoma, as well as endometrial, ovarian, head and neck, thyroid, colon, and breast cancers (Fig. 3a, b, Supplementary Figure 4, and Supplementary Table 1). Virus-specific T cells identified in tumors were phenotypically different (expressing CD69 and CD103, markers associated with resident memory T cells^14^) compared to those found in blood, indicating that these were not blood contaminates and constituted T cells within the tumor (Fig. 3a). These results extend recent observations of virus-specific T cells in colorectal and lung cancers^15^.

To test if antiviral T cells in human tumors could respond to peptide therapy, we first stimulated freshly isolated T cells from human tumors with viral peptides ex vivo and measured cytokine expression 12 h later. We observed a measurable increase in TNFα and IFNγ production, indicating that both mouse and human intratumoral T cells are capable of being triggered by viral peptides (Supplementary Figure 5). To test if reactivated peptide therapy could promote tumor-wide immune activation in situ, we employed an organotypic ex vivo slice culture model of fresh human tumors. This method allows for preservation of cellular composition and tissue architecture of the tumor^16^. Blood from HLA-A*02 patients was screened for tetramer + cells, which then informed the cocktail of viral-derived peptides to add to tumor sections obtained from that same patient. RNAseq was performed on tumor slices 9 h after peptide addition and compared to control cultures (Fig. 3c, Supplementary Table 2). In three out of four human tumors, there was upregulation of genes involved in immune activation. Further analysis showed highly similar patterns of enriched upstream transcriptional regulators in human ex vivo, mouse ex vivo and mouse in vivo tumors (Fig. 3d). Importantly, these proof-of-principle data demonstrate that human antiviral T cells are capable of reactivating within tumors to trigger immune activation.

### Viral peptides sensitize B16 melanoma to checkpoint blockade

**Fig. 4:**
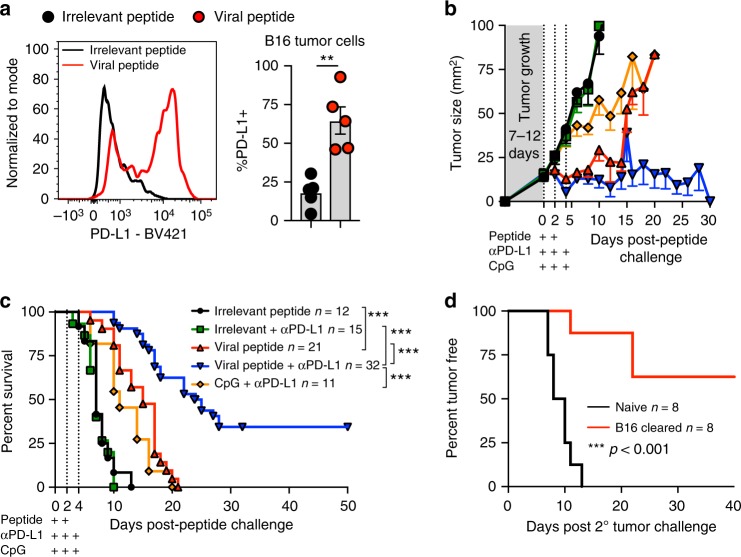
Antiviral T-cell reactivation sensitizes B16 to checkpoint blockade therapy. **a** PD-L1 expression on B16 tumor cells 24 h following intratumoral irrelevant (black circles) or viral peptide (red circles), *n* = 5; data pooled from two experiments. Lines represent means and error bars are SEM. **b**, **c** Tumor growth (**b**) and survival (**c**) of OT-I immune chimeras with B16 treated with irrelevant peptide (black lines), irrelevant peptide with anti-PD-L1 (green lines), viral peptide (red lines), viral peptide with anti-PD-L1 (blue lines) or CpG with anti-PD-L1 (orange lines). Peptide was injected intratumorally twice 24 h apart, CpG was injected intratumorally thrice 24 h apart, and anti-PD-L1 antibody was delivered i.v. thrice 24 h apart, as denoted by plus symbols. Pooled data from at least two experiments. **d** Percent of naive control (black lines) or cured peptide + anti-PD-L1 treated mice that remained tumor-free after B16 re-challenge in opposite flank (red lines); data pooled from two experiments. Sample size for tumor growth and survival plot indicated in the figures where *n* = number of mice. Statistical significance was determined by unpaired two-tailed *t*-test (**a**) and Log-rank Mantel–Cox test (**c**, **d**) where **p < 0.01, ***p < 0.001

Patients with PD-L1-positive tumors are typically more responsive to checkpoint blockade^1^. While mouse B16 melanoma is refractory to checkpoint blockade therapy^17^, when we stained for PD-L1 we observed that it was upregulated on tumor cells after peptide therapy (Fig. 4a). This prompted us to test whether PD-L1 blockade would now be effective in the setting of peptide therapy. OT-I chimeras received B16 melanoma. Seven to twelve days later, palpable tumors were injected twice or thrice with reactivating viral SIINFEKL peptide or control irrelevant peptide in conjunction with three injections of anti-PD-L1 antibody i.v. In this case, we observed complete eradication of B16 in 34% of mice (Fig. 4b, c, and Supplementary Figure 6). Of note, combinatorial peptide and checkpoint blockade therapy was far more effective than anti-PD-L1 combined with a TLR agonist that is currently in clinical trials, CpG (Fig. 4b, c and Supplementary Figure 6)^18^. When peptide therapy-cured mice were subsequently challenged > 5 weeks later on the opposite flank with B16 melanoma (in the absence of additional peptide therapy or any other treatments), these tumors failed to grow in most mice (Fig. 4d). This indicates that systemic anti-tumor immunity had been established.

## Discussion

This study highlights that human tumors from diverse cancers commonly contain antiviral CD8+ T cells, consistent with reports that many tumor infiltrating lymphocytes may not be tumor-specific, and extending recent work focused on colorectal and lung cancer^15,19,20^. This merits consideration given that the frequency of CD8 + (and CD103 +) tumor infiltrating lymphocytes correlates with favorable prognosis^21–24^. We go on to show that we can repurpose these antiviral memory T cell as a tumor therapy.

Major impediments to immune control over tumors are the immunosuppressive microenvironment and the exhaustion of tumor-specific T cells^3^. Overcoming this immunosuppression and inducing a durable systemic tumor-specific immune response is the ultimate goal of tumor immunotherapy. The peptide therapy described in this study operates by ‘tricking’ antiviral T cells into perceiving a local reinfection that broadly activates innate and adaptive immunity. Inducing an immunostimulatory environment within tumors is an active area of study and notable therapies being pursued are TLR agonists and oncolytic viruses. While not directly compared beyond impacts on tumor growth (Fig. 4), it is likely there are mechanistic differences between peptide therapy, which activates cytotoxic CD8 T cells to perform a broad array of immune functions, and innate-activating TLR agonists. Adaptive immunity requires significantly more levels of regulation and danger signals to respond because once the response has been established, future activity occurs with a sensitive trigger, effector functions are much more potent, and participating populations can undergo massive self-amplification. Moreover, this hyper-responsive state is maintained long-term due to durable T-cell memory. Peptide therapy bypasses the need for vaccination and taps into adaptive cytotoxic memory T-cell populations that have already passed through these regulatory checkpoints in order to control pathogens. When established antiviral memory CD8+ T cells are triggered by peptide, they execute a broad array of functions that include cytotoxicity and the activation of numerous pathways within the innate and adaptive immune system.

Oncolytic virotherapies have shown exciting promise and while originally used as a targeted way to kill tumor cells, this therapy also induces anti-tumor immunostimulatory effects. Because of the nature of the therapy (a live replicating virus), induction of an antiviral immune response can work to inhibit the therapy. Additionally, there are viruses currently being tested, such as herpesviruses, poliovirus and measles virus, that may stimulate pre-existing immunity. Presence of antibodies specific to the virotherapy can pose a challenge as this can inhibit infection and potentially limit efficacy^25^. Despite these limitations, clinical trials have demonstrated promising efficacy, in particular with T-VEC, an oncolytic herpesvirus that has demonstrated efficacy against melanoma when injected intratumorally^26,27^. While not tested, an intriguing possibility is that some current virotherapies may be tapping into pre-existing T-cell immunity specific to those viruses which may exist in tumors.

Immunotherapies that more directly work to induce an anti-tumor T-cell response have also shown exciting promise and include vaccines targeting tumor antigens, checkpoint blockade, and adoptive cell therapies including chimeric antigen receptor (CAR) T-cell therapy, however, clinical efficacy against solid tumors remains suboptimal or does not work for many patients^4^. Viral peptide therapy may synergize with these existing therapies, for example by making ‘cold’ tumors ‘hot’ (recruiting T cells), extending the range of patients that respond to PD-L1 or CTLA-4 checkpoint blockade, or by recruiting ACT to solid tumors. A potential disadvantage is that viral peptides may have to be matched to patient HLA type. HLAs, however, are easy to assess, are shared by large populations, and peptides from ubiquitous human pathogens have already been defined for common HLAs. Additionally, these issues may be counterbalanced by safety and manufacturing advantages as relevant synthetic viral peptides are only 8–10 amino acids. Moreover, peptide therapy requires no adjuvant, vaccine, or replicating agent potentially subject to risky safety profiles or issues with dosing and tropism, nor costly identification of tumor-specific antigens that may not be immunogenic. In summary, re-stimulating known antiviral immunity via defined peptides from common pathogens provides a unique therapeutic avenue for cancer immunotherapy.

## Methods

### Mice

C57BL/6J (B6) female mice were purchased from The Jackson Laboratory (Bar Harbor, ME) and were maintained in specific-pathogen-free conditions at the University of Minnesota. *BRaf*^*CA*^*,Pten*^*loxP*^*,Tyr::Cre-ER*^*T2*^ male and female mice were obtained from the Jackson Laboratory and bred in our animal colony. CD90.1^+^ OT-I and CD45.1^+^ OT-I mice were fully backcrossed to C57BL/6J mice and maintained in our animal colony. Sample size was chosen on the basis of previous experience. No sample exclusion criteria were applied. No method of randomization was used during group allocation, and investigators were not blinded. All mice used in experiments were 5–14 weeks of age. All mice were used in accordance with the Institutional Animal Care and Use Committees guidelines at the University of Minnesota.

### Adoptive transfers and infections

We generated OT-I immune chimeras by transferring 5 × 10^4^ CD90.1 or CD45.1 OT-I CD8^+^ T cells from female mice into naive 6–8-week-old C57BL/6J female mice, and then infecting those mice with 1 × 10^6^ PFU of vesicular stomatitis virus expressing chicken ovalbumin (VSVova) i.v. Alternatively, 5 × 10^4^ CD90.1 or CD45.1 OT-I CD8^+^ T cells from female mice were injected i.v. into 5-week-old male or female *BRaf*^*CA*^*,Pten*^*loxP*^*,Tyr::Cre-ER*^*T2*^ mice which were then infected with 5 × 10^5^ PFU of VSV-OVA i.v. For endogenous CD8^+^ T-cell studies, we infected naive C57BL/6J mice with 10^6^ PFU of VSV strain Indiana i.v. followed by Vaccinia virus expressing the N epitope from VSV i.v. 30 days later. Lymphocytes were stained with H-2K^b^/B8R and H-2K^b^/N MHC I tetramers.

### Lymphocyte isolation and phenotyping of mouse cells

We used an intravascular staining method to discriminate cells present in the vasculature from cells in the tissue parenchyma, as described^28^ for 12-h timepoints. In brief, we injected mice i.v. with biotin/fluorochrome-conjugated anti-CD45 i.v. Three minutes after the injection, we euthanized the animals and harvested tissues as described^29^. B16 tumors were removed and processed by gentleMACS Dissociator. Skin was digested in Collagenase IV (Sigma) for 1 h then dissociated via gentleMACS Dissociator (Miltenyi Biotec) twice. Isolated mouse cells were stained with antibodies to CD11b (clone M1/70, BD Biosciences, 561114), MHCII I-A/I-E (clone M5/114.15.2, BioLegend, 107635), CD86 (clone GL1, BD Biosciences, 563055), CD11c (clone N418, BioLegend, 117311), CD103 (clone 2E7, eBioscience, 17-1031-80), CD45 (clone 30-F11, BD Biosciences, 563709), CCR7 (clone 4B12, eBioscience, 12-1971-82), NK1.1 (clone PK136, BioLegend, 108728), CD3 (clone 145-2C11, BD Biosciences, 563004), CD8a (clone 53-6.7, BioLegend, 100743), IFNγ (clone XMG1.2, BD Biosciences, 54411), CD25 (clone PC61, BD Biosciences, 557192), CD44 (clone IM7, BioLegend,103059), granzyme B (clone GB11, Invitrogen, GRB04), CD69 (clone H1.2F3, BD Biosciences, 562455), MHCI H-2K^b^/H-2D^b^ (clone 28-8-6, BioLegend, 107635), CD274 PD-L1 (clone 10F.9G2, BioLegend 124315), gp100 (clone EP4863, Abcam, ab137078), and anti-rabbit AF647 (Invitrogen, A-21245). All cells were stained at antibody dilutions of 1:100 except for granzyme B (1:50), MHCII I-A/I-E (1:400), gp100 (1:1000), and anti-rabbit Ig (1:400). Cells stained intracellularly (for IFNγ, granzyme B and gp100) were permeabilized using Tonbo or ebioscience Fixation/permeabilization kits. Cell viability was determined with Ghost Dye 780 (Tonbo Biosciences). Gating strategy shown in Supplementary Figure 7. Enumeration of cells was done using PKH26 reference microbeads (Sigma). The stained samples were acquired with LSRII or LSR Fortessa flow cytometers (BD) and analyzed with FlowJo software (Treestar).

### Immunofluorescence microscopy

B16 tumors were harvested, then fixed in 2% paraformaldehyde for 2 h before being treated with 30% sucrose overnight for cryoprotection. The sucrose-treated tissue was embedded in OCT tissue-freezing medium and frozen in an isopentane liquid bath. Frozen blocks were processed, stained, and imaged including staining with antibodies to CD8-β (YTS156.7.7; BD Biosciences), CD90.1 (OX-7; BD Biosciences), and CD45.1 (A20; Biolegend). We also counterstained with 4′,6-diamidino-2-phenylindole dihydrochloride (DAPI) to detect nuclei.

### Tumor models and treatment

150,000 B16F10 or 500,000 MC38 cells were injected i.d. into the right flank (or 50,000 B16 in left flank for re-challenge experiment). Tumor growth was monitored and mice were euthanized upon reaching endpoint criteria of > 10 mm in one dimension or ulceration. Tumor size was calculated by multiplying length × width (mm^2^). B16 and MC38 cells were maintained in RPMI 1640 or DMEM, respectively, supplemented with 10% FBS, L-glutamine, sodium pyruvate, penicillin/streptomycin, HEPES, nonessential amino acids, and beta-mercapto-ethanol. Alternatively, tamoxifen (4-hydroxytamoxifen) Sigma was diluted in DMSO at 8 mg/ml and 10 µl was injected intradermally into the right flank of *Braf/Pten* mice. *Braf/Pten* mice that had observable spontaneous tumors at the time of tamoxifen injection were excluded. *Braf/Pten* mice that developed spontaneous tumors after 4-OHT delivery were included, however, all measurements were taken from the induced tumor. For local tumor T cell reactivation experiments involving peptides, 0.5 µg of the indicated peptides (New England Peptides) were delivered intratumorally or into the skin via tattoo gun (Fig. 2 and Supplementary Figure 2) or by direct intratumor injection in a volume of 30 µl. Alternatively, CpG ODN1826 (Invivogen) was injected intratumorally at a dose of 10 μg in 30 µl. Anti-PD-L1 (clone B7-H1, Bioxcel) was injected i.v. at 0.2 mg/mouse every other day for a total of three times starting at the time of first intratumoral peptide injection. Peptides used in mouse studies: KAVYNFATM (gp33) from LCMV (used as control irrelevant); TSYKFESV (B8R) from Vaccinia virus; RGYVYQGL (N) from VSV; and SIINFEKL from ovalbumin.

### Procurement and processing of human blood and tissue samples

All tumor tissue and blood was obtained from male or female patients age 16–80 undergoing routine surgical resection of solid tumors or tumor metastases. Tumor tissue not required for pathological diagnostic procedures was obtained after surgical resection at the University of Minnesota and collected and de-identified by the Tissue Procurement Facility (BioNet, University of Minnesota). Informed consent was obtained from all subjects. The University of Minnesota Institutional Review Board approved all protocols used. Blood was collected in EDTA collection tubes and tumors were collected in RPMI media containing 5% FBS. All samples were stored at 4 °C until processed (within 24 h). Specimens reported on were obtained from HLA*A02 + patients that had sufficient tetramer + cells for analysis by flow cytometry. Human blood was processed by Ficoll gradient. Tumors were minced and digested in Collagenase type IV (endometrial) or Collagenase Type I (all others). They were then dissociated via gentleMACS Dissociator 1 × (glioblastoma or brain metastases) or 2 × (all others) and lymphocytes purified on a 44/67% Percoll (GE Healthcare) gradient. Lymphocytes were stained for anti-human HLA-A2 (clone BB7.2, BioLegend, 343324), CCR7 (clone G043H7, BioLegend, 353208), CD45RO (clone UCHL1, BioLegend, 304232), CD8α (clone SK1, BD Biosciences, 561945), CD3e (clone SP34-2, BD Biosciences, 557917), CD4 (clone L200, BD Biosciences, 551980), CD69 (clone FN50, BioLegend, 310926), CD103 (clone HML-1, Beckman Coulter, IM1856U), IFNγ (clone B27, BD Biosciences, 554700), TNFα (clone Mab11, BD Biosciences, 554514). Cells were stained at antibody dilutions of 1:30. Samples were also stained for HLA-A*02 tetramers (made in house) for EBV_GLC_, EBV_CGL_, CMV_NLV_, and Flu_GIL_. Viability was assessed by live/dead staining with GhostDye510 (Tonbo biosciences). Gating strategy shown in Supplementary Figure 8. For in vitro stimulation, isolated lymphocytes were incubated with viral peptides (10 μg/mL) or control DMSO in RPMI media containing brefeldin A (GolgiPlug, BD Biosciences), monensin (GolgiStop, BD Biosciences), 10% FBS, L-glutamine, sodium pyruvate, penicillin/streptomycin, HEPES, and nonessential amino acids. Cultures were incubated overnight and stained for the above antibodies/reagents for flow cytometry. Peptides used in human studies: CLGGLLTMV (EBV_CLG_), GLCTLVAML (EBV_GLC_), NLVPMVATV (CMV_NLV_), GILGFVFTL (Flu_GIL_).

### Transwell cultures and RNA isolation

Tumors were sliced into thin sections manually with a sharp surgical blade. Sections were then incubated in RPMI media containing 10% FBS, L-glutamine, sodium pyruvate, penicillin/streptomycin, HEPES, nonessential amino acids, and beta-mercapto-ethanol on 12-well polycarbonate transwell inserts with a 0.4 μm pore size (Corning) and maintained in 5% CO_2_ and atmospheric oxygen levels^16^. Tissues were incubated with viral peptides at 10 μg/mL or in equal volume of DMSO for 9 h. Tissues with poor viability after culture were excluded. Tumor sections were stored in RNAlater (ThermoFisher) at 4 °C overnight, then stored at −80 until further processing. For RNA isolation, tissue was thawed on ice in 1 mL TRIZOL (Invitrogen) then homogenized with a Tissue Tearor homogenizer, BioSpec. RNA was then isolated following the TRIZOL recommended protocol. Resulting RNA was then further purified using Qiagen RNA Cleanup Kit.

### RNA library preparation and sequencing

mRNA libraries were generated using the TruSeq Stranded mRNA Library Prep kit (Illumina) and sequenced on an Illumina HiSeq 2500 in 50-base paired-end reactions. Fastq files were verified for quality control using the fastqc software package. Low-quality segments and adapters were trimmed using Trimmomatic. Quality-filtered reads were aligned to either the mouse genome GRCm38 or the human genome GRCh38 using Hisat2^30^. Differentially expressed genes were determined using the DESeq2 R package^31^ where false-discovery rate (FDR) < 0.1 was considered significant. Upstream transcriptional regulators were generated through the use of IPA (QIAGEN Inc., https://www.qiagenbioinformatics.com/products/ingenuity-pathway-analysis)^32^.

### Statistics

Data were subjected to the Shapiro–Wilk normality test to determine whether they were sampled from a Gaussian distribution. If a Gaussian model of sampling was satisfied, parametric tests (unpaired two-tailed Student’s *t*-test for two groups and one-way ANOVA with Bonferroni multiple comparison test for more than two groups) were used. If the samples deviated from a Gaussian distribution, non-parametric tests (Mann–Whitney U test for two groups, Kruskal–Wallis with Dunn’s multiple comparison test for more than two groups) were used. All statistical analysis was done in GraphPad Prism (GraphPad Software Inc.). *p* < 0.05 was considered significant.

### Reporting summary

Further information on experimental design is available in the Nature Research Reporting Summary linked to this article.

## Supplementary information

Supplementary Information

Reporting Summary

## Data Availability

RNAseq data are deposited to the GEO databaseunder the accession code GSE124620.
