# Supplementary material for: Virus-specific memory T cells populate tumors and can be repurposed for tumor immunotherapy
Source: Nat Commun. 2019 Feb 4;10:567. doi: 10.1038/s41467-019-08534-1 (PMC6362136; doi:10.1038/s41467-019-08534-1)
Supplement: Supplementary file 1 — Supplementary Information [file 41467_2019_8534_MOESM1_ESM.pdf]

Virus-specific memory T cells populate tumors and can be repurposed for tumor immunotherapy

Rosato et. al.

Supplementary Information

Supplementary Figure 1

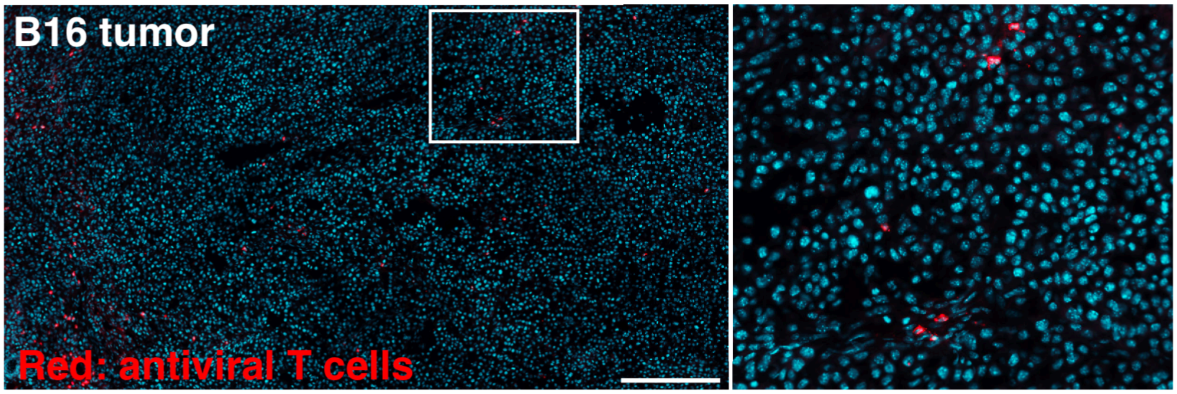

Supplementary Figure 1. Antiviral memory T cells populate B16 tumors. Representative immunofluorescence staining of *Braf/Pten* tumor. Red, OT-I; teal, 4',6-diamidino-2-phenylindole (DAPI)-stained nuclei. Scale bar=250um.

Supplementary Figure 2

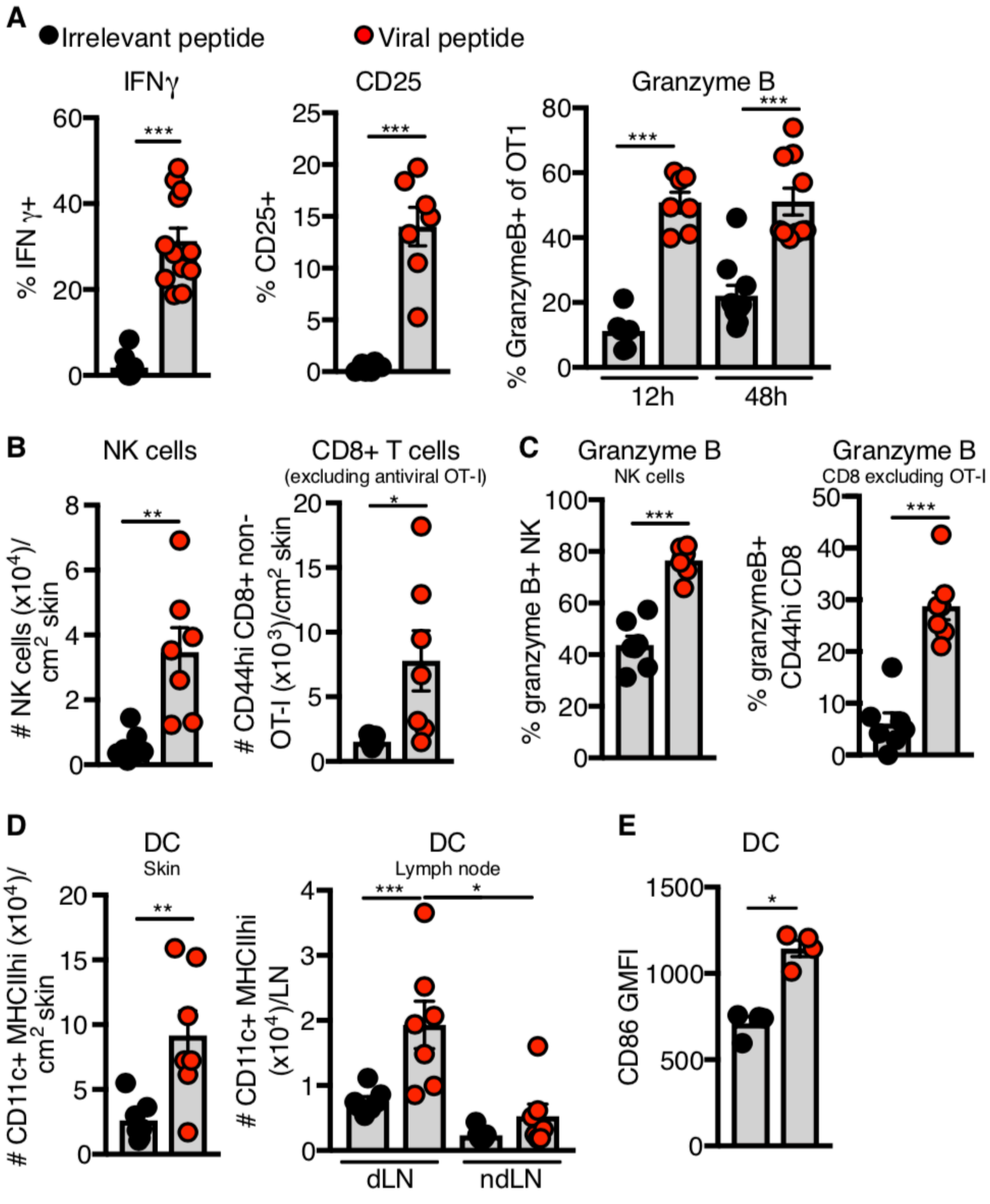

Supplementary Figure 2. OT-I reactivation in the skin mediates immune activation and recruitment. A) Proportion of IFNγ+, CD25+ and granzyme B+ OT-I in skin after exposing skin to control irrelevant (black dots) or viral SIINFEKL peptide (red dots). IFNγ and granzyme B 12h: n=10 mice (irrelevant), n=12 mice (viral); CD25: n=6 mice (irrelevant), n=7 mice (viral), granzyme B 48h: n=10 mice. **B)** Quantification of NK cells and non-viral peptide specific CD44hi CD8+ T cells in skin 48hrs post-peptide (n=7mice). **C)** Frequency of granzyme B+ NK and non-viral peptide specific CD8+ T cells in skin 48h post-peptide (n=7 mice). **D)** Quantification of CD11c+ MHCIIhi DC in the skin (left) and skin-draining (dLN) and non-draining (ndLN) lymph node 48hrs post-peptide (n=7 mice). **E)** Geometric mean fluorescence intensity of CD86 in CD11c+ MHCIIhi DC in skin (n=4 mice, representative experiment). Significance was determined by unpaired two-tailed Mann-Whitney test for **A, C (**CD8), **and E;** unpaired two-tailed t-test for **B**, **C** (NK), **D** (skin); and one-way unpaired Kruskal-Wallis ANOVA with Dunn's post-hoc test for **D** (LN). All data are pooled from at least 2 independent experiments. Lines represent means and error bars are SEM. *p<0.05, **p<0.01, ***p<0.001.

Supplementary Figure 3

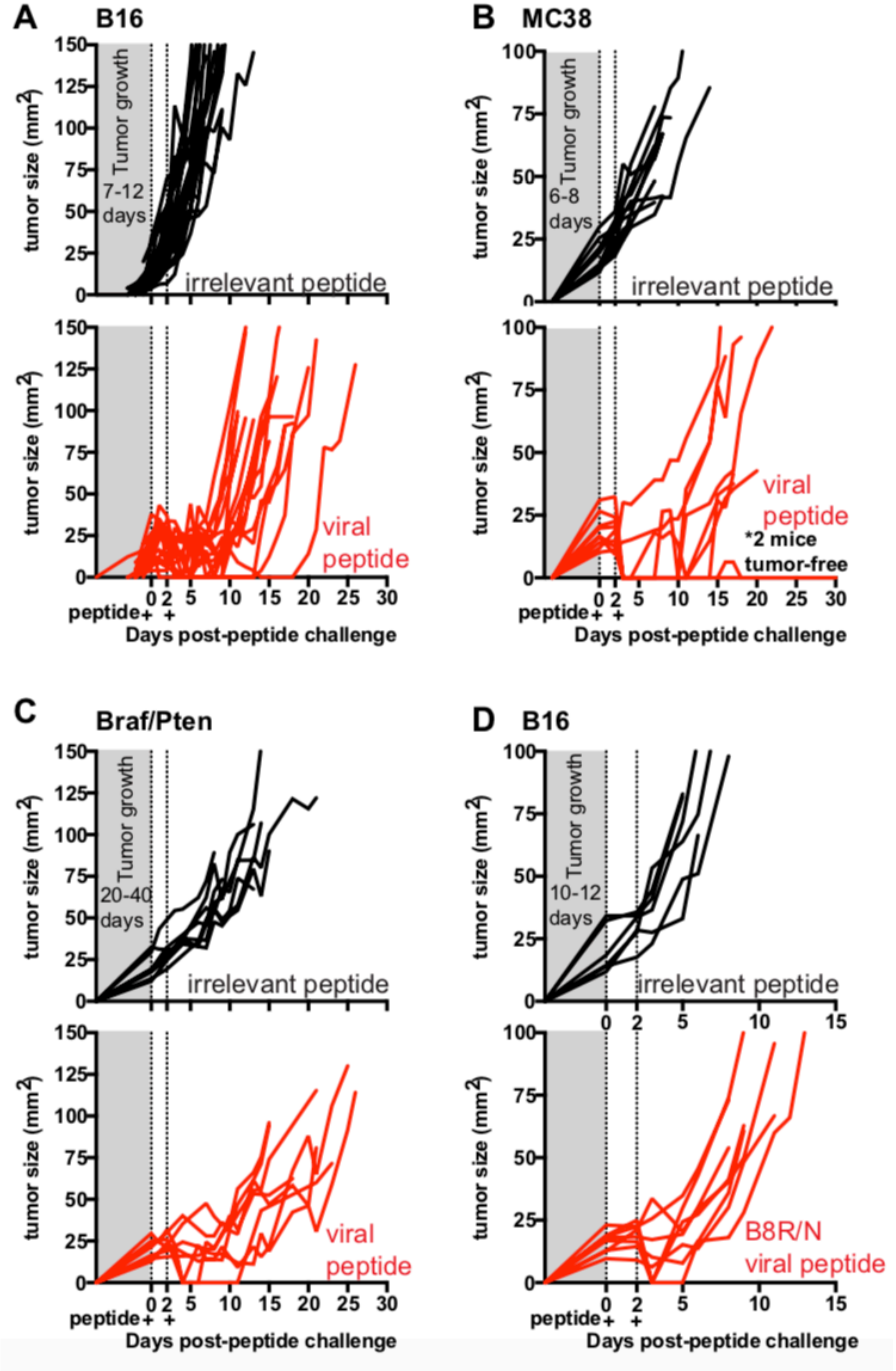

Supplementary Figure 3. Antiviral memory cell activation arrests tumor growth. Related to Figure 1d-g. Tumor growth of individual mice following two intratumoral peptide injections in OT-I chimeras with B16 melanoma (data from 3 experiments) **(D),** OT-I chimeras with MC38 (pooled data from 2 experiments) **(E),** *Braf/Pten* OT-I chimeras (data from 3 experiments) **(F)**, or mice with endogenous memory generated to VSV Indiana and VV-N (data from 2 experiments) **(G).** As indicated, black lines denote irrelevant control peptide and red lines denote viral peptide in figures. Each line represents one individual mouse. Average tumor growth depicted in figures 1d-g.

Supplementary Figure 4

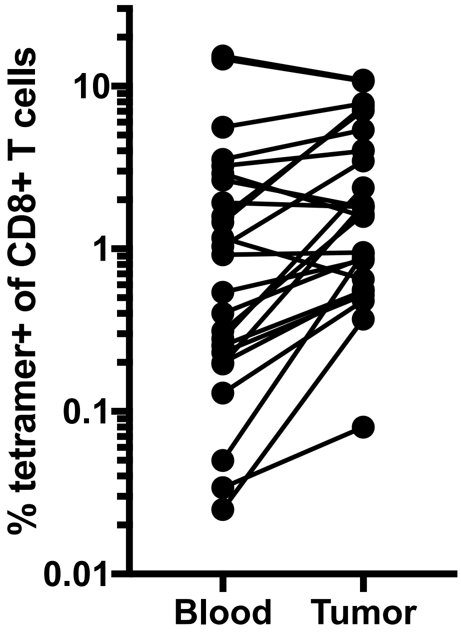

Supplementary Figure 4. Frequency of EBV, CMV and Flu tetramer+ CD8+ T cells in blood and tumor. Sum of the frequencies of EBV_{GLC}, EBV_{CLG}, CMV_{NLV} and Flu_{GIL} tetramer+ cells in blood and all tumors combined. Each symbol represents a patient.

Supplementary Figure 5

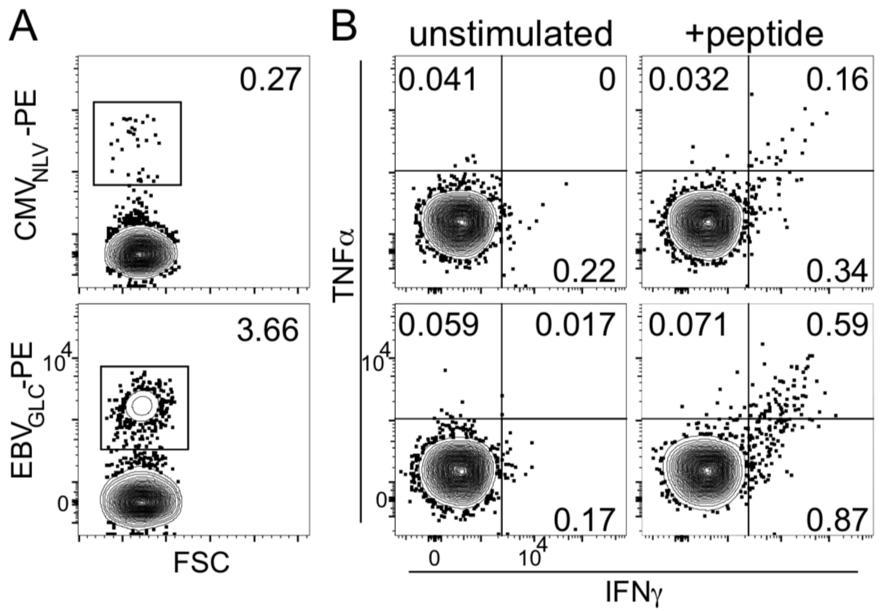

Supplementary Figure 5. Antiviral T cells isolated from human tumors reactivate to produce pro-inflammatory cytokines. a, Frequency of tetramer+ cells isolated from a human endometrial tumor prior to stimulation. Gated on CD3+/CD8+. **b**, Frequency of total CD8+/CD3+ cells expressing TNFα and IFNγ after *in vitro* culture with control or EBV_{GIL} and CMV_{NLV} peptides.

Supplementary Figure 6

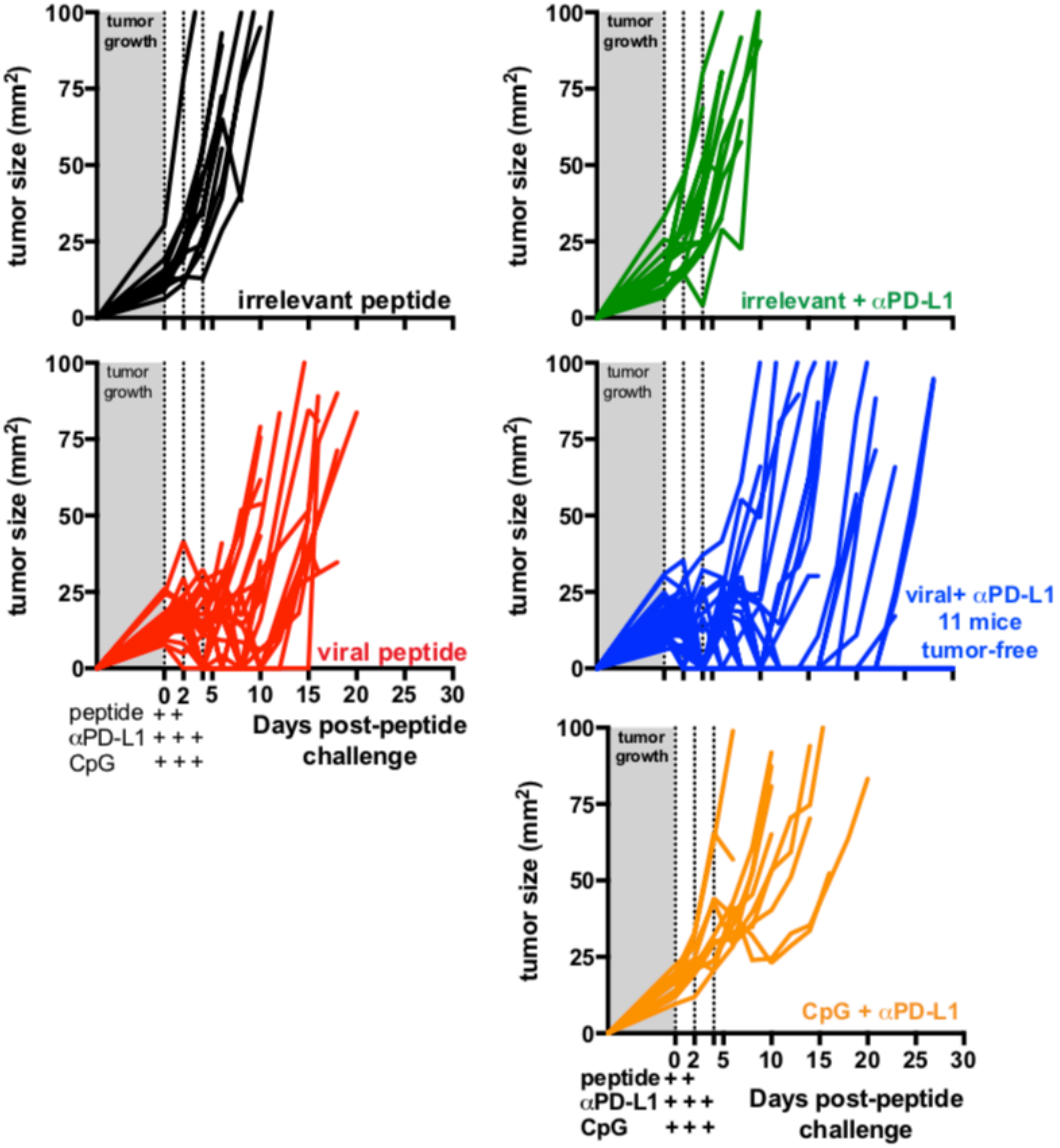

Supplementary Figure 6. Tumor growth in individual mice. Related to Figure 4b. Tumor growth of individual OT-I immune chimeras with B16 treated with irrelevant peptide (black lines), irrelevant peptide with anti-PD-L1 (green lines), viral peptide (red lines), viral peptide with anti-PD-L1 (blue lines) or CpG with anti-PD-L1 (orange lines). Peptide was injected intratumorally twice 24h apart, CpG was injected intratumorally thrice 24h apart, and anti-PD-L1 antibody was delivered i.v. thrice 24h apart, as denoted by plus symbols. Pooled data from at least 2 experiments. Each line represents one individual mouse. Average tumor growth depicted in figures 1d-g.

Supplementary Figure 7

A

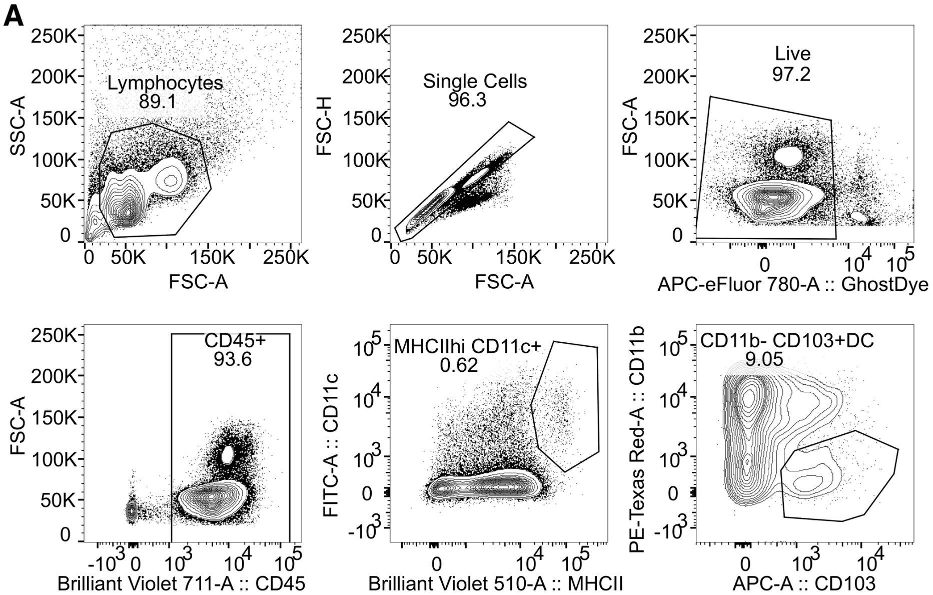

B

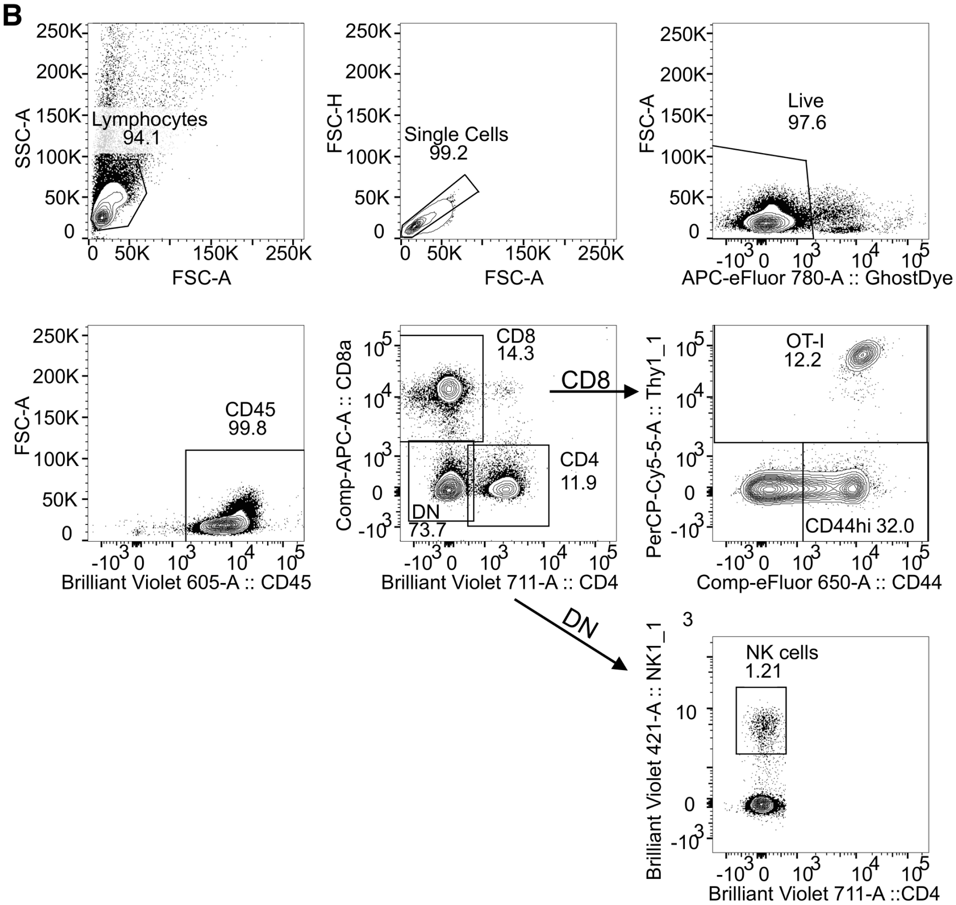

Supplementary Figure 7. Gating strategy for mouse tissue. Related to Figures 1, 2 and 4. Starting cells were gated by FSC/SSC gates and then with viability dye (GhostDye) to select live population. **a,** Cells were further gated on CD45+ then MHCIIhi/ CD11c+ and finally CD11b-/CD103+ to examine CD103+ DCs. Expression of CD86 and CCR7 was examined as indicated in the figure legends. **b,** To examine virus-specific CD8 T cells, cells were gated on CD8+/CD4- and then on the congenic marker (CD90.1 or CD45.1). Gating of congenic marker-negative, CD44hi cells was done to examine non-peptide specific memory T cells. CD8-/CD4- cells were further gated on NK1.1+ to examine NK cells. Expression of IFNγ, CD25 and granzyme B was examined. Example shown is from the tumor-draining lymph node of a *Braf/Pten* mouse.

Supplementary Figure 8

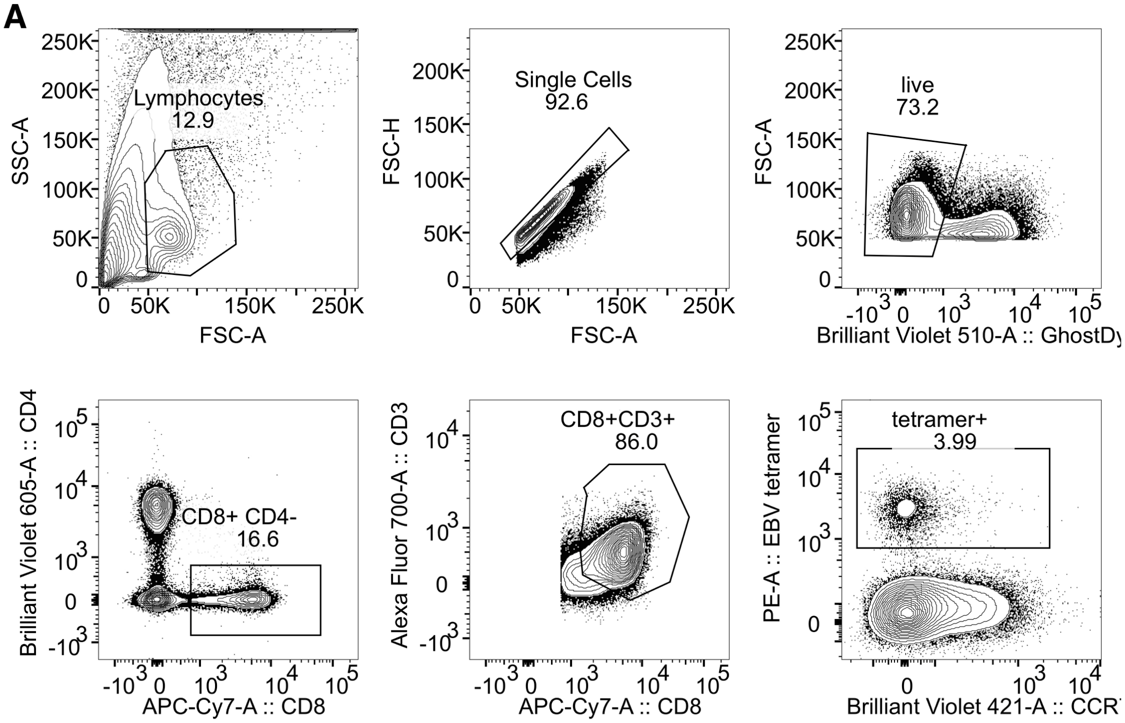

Supplementary Figure 8. Gating strategy for human tissue. Related to Figure 3. Starting cells were gated by FSC/SSC gates and then with viability dye (GhostDye) to select live population. These cells were further gated on CD8a+/CD4- then CD3+, and tetramer was gated on to focus on virus-specific CD8+ T cells. Expression of CD69, CD103, CCR7, IFNγ and TNFα was examined on these populations as indicated in the figure legends. Example shown is an endometrial tumor.

Supplementary Table 1

Study ID	Tissue	% of CD8+ T cells CMV (NLV)	EBV (GLC)	EBV (CLG)	Flu (GIL)	Sum
**Endometrial**						
TC17_002	Endometrial carcinoma	n/a	3.99	n/a	n/a	3.99
	Blood	0	3.23	0	0	3.23
TC17_007	Endometrial carcinoma	0.27	3.66	n/a	n/a	3.93
	Blood	3.28	0.26	0	0	3.54
TC17_006	Endometrial carcinoma	n/a	0.48	n/a	n/a	0.48
	Blood	0	0.13	0	0	0.13
TC17_011	Endometrial carcinoma	n/a	0.54	0.06	0.047	0.65
	Blood	0	0.73	0.37	0.069	1.17
TC17_014	Endometrial carcinoma	0.17	0.24	0.11	0.03	0.55
	Blood	0.11	0.022	0.057	0.065	0.25
T17_1404	Endometrial carcinoma	n/a	0.62	0.33	n/a	0.95
	Blood	0	0.68	0.24	0	0.92
T17_1424	Endometrial carcinoma	0.16	6.95	n/a	n/a	7.11
	Blood	1.09	0.49	0	0	1.58
T17_015	Endometrial carcinoma	0.14	1.37	0.23	0.073	1.81
	Blood	1.02	0.76	0.085	0.056	1.92
T18_0241	Endometrial carcinoma	0.56	1.84	0.92	0	3.32
T18_0286	Endometrial carcinoma	0	3.44	0.054	0.16	3.65
	Blood	0	7.8	0.081	0.048	7.93
**Head and neck**						
T17_0798	Tongue SCC	0.22	0	10.7	0	10.92
	Blood	15.2	0	0.14	0	15.34
T17_0746	Floor of Mouth SCC	0	0	0.52	0	0.52
	Blood	0	0	0.23	0	0.23
T17_1293	Tongue SCC	0	5.08	1.96	0.45	7.49
	Normal adjacent tongue	0	2.11	n/a	n/a	2.11
	Blood	0	0.94	0.45	0.072	1.46
T17-1350	Tongue SCC	0.31	1.27	n/a	n/a	1.58
	Blood	2.76	0.12	0.02	0	2.90
**Renal**						
T17_0740	Renal cell carcinoma	0.85	0	0	0	0.85
	Normal adjacent kidney	0.39	0	0	0	0.39
	Blood	0.54	0	0	0	0.54
T17_0820	Renal cell carcinoma	0	0.25	0.12	0	0.37
	Normal adjacent kidney	0	0.41	0.098	0	0.51
	Blood	0	n/a	0.025	0	0.03
T17_1040	Renal cell carcinoma	0	0.8	0.079	0	0.88
	Blood	0	0.29	0.11	0	0.40
T18_0279	Renal cell carcinoma	0.67	0	0	0	0.67
	Normal adjacent kidney	2.33	0	0	0	2.33
**Breast**						
T17-1078	Breast tumor	2.5	5.33	0	0	7.83
	Normal adjacent breast	1.84	3.45	0	0	5.29
	Blood	5.23	0.36	0	0	5.59
T17_1152	Breast tumor	0.11	3.24	0	0.13	3.48
	Normal adjacent breast	n/a	2.6	0	n/a	2.60
	Blood	0.07	0.92	0	0.046	1.04
**Glioblastoma**						
T17_1298	Glioblastoma multiforme	0	0.36	0	1.49	1.85
	Blood	0	0.076	0	0.12	0.20
T17_1321	Glioblastoma multiforme	0	0	0	0.93	0.93
	Blood	0	0	0	0.05	0.05
T18_0128	Glioblastoma multiforme	0	n/a	n/a	0.56	0.56
	Blood	0	0.036	0.033	0.13	0.20
T18_0137	Glioblastoma multiforme	4.71	4.2	0.32	1.53	10.76
	Blood	12.7	1.54	0.11	0.39	14.74
T18_0350	Glioblastoma multiforme	0	3	0.54	0.56	4.10
	Blood	0	2.75	0.083	0.045	2.88
T18_0358	Glioblastoma multiforme	0	0	0	3.37	3.37
	Blood	0	0	0	0.12	0.12
**Brain metastases**						
T17_0844	Brain met from NSCLC	0	0.079	0.32	3.65	4.05
T18_0060	Brain met from breast	0	1.3	0.33	0	1.63
	Blood	0	0.092	0.22	0	0.31
T18_0334	Brain met from melanoma	n/a	0.29	0.046	0.07	0.41
	Blood	0	0.11	0.039	0.02	0.17
**Other**						
T17_0864	Adenocarcinoma	0	2.38	0	0	2.38
	Blood	0	0.28	0	0	0.28
T17_1136	Thyroid- Hurthle cell	1.29	0.4	0.11	n/a	1.80
	Blood	0.92	1.65	n/a	0.081	2.65
T17_0750	Myxofibrosarcoma	0	0	0.077	0	0.08
	Blood	0	0	0.034	0	0.03
T18_0237	Colorectal cancer	0	1.87	0	0	1.87
	Blood	0	0.25	0	0	0.25

% of CD8s	
(red)	>10
(dark orange)	2-10
(orange)	0.5-2
(yellow)	0.1-0.5
(pale yellow)	0.02-0.1
(white)	0 or n/a

Supplementary Table 1. Summary of the frequency of virus-specific CD8+ T cells in human tumors and paired blood. Abbreviations: SCC, squamous cell carcinoma; NSCLC, non-small cell lung carcinoma; n/a, did not perform analysis or not enough cells to determine frequency.

Supplementary Table 2

Study ID	Tumor type	%CMV (NLV)	%EBV (GLC)	%EBV (CLG)	%Flu (GIL)	Order in Fig. 3d
T18_0241	endometrial	0.56	1.84	0.92	x	1
T17_1424	endometrial	0.16	6.95	x	x	2
T18_0237	colon	x	2.14	x	x	3
T18_0286	endometrial	x	3.44	x	0.16	not included

Supplementary Table 2. Human tumors used for RNAseq in Fig 3d. Frequency of T cells specific for peptides used in cultures. Gated on CD3+/CD8+.
