# Supplementary material for: Virus-specific memory T cells populate tumors and can be repurposed for tumor immunotherapy
Source: Nat Commun. 2019 Feb 4;10:567. doi: 10.1038/s41467-019-08534-1 (PMC6362136; doi:10.1038/s41467-019-08534-1)
Supplement: Supplementary file 2 — Reporting Summary [file 41467_2019_8534_MOESM2_ESM.pdf]

nature research

Corresponding author(s): David Masopust; NCOMMS-18-35324A

Last updated by author(s): Dec 27, 2018

Reporting Summary

Nature Research wishes to improve the reproducibility of the work that we publish. This form provides structure for consistency and transparency in reporting. For further information on Nature Research policies, see Authors & Referees and the Editorial Policy Checklist.

Statistics

For all statistical analyses, confirm that the following items are present in the figure legend, table legend, main text, or Methods section.

n/a	Confirmed	
☐	☒	The exact sample size (*n*) for each experimental group/condition, given as a discrete number and unit of measurement
☐	☒	A statement on whether measurements were taken from distinct samples or whether the same sample was measured repeatedly
☐	☒	The statistical test(s) used AND whether they are one- or two-sided<br>*Only common tests should be described solely by name; describe more complex techniques in the Methods section.*
☒	☐	A description of all covariates tested
☐	☒	A description of any assumptions or corrections, such as tests of normality and adjustment for multiple comparisons
☐	☒	A full description of the statistical parameters including central tendency (e.g. means) or other basic estimates (e.g. regression coefficient) AND variation (e.g. standard deviation) or associated estimates of uncertainty (e.g. confidence intervals)
☒	☐	For null hypothesis testing, the test statistic (e.g. *F*, *t*, *r*) with confidence intervals, effect sizes, degrees of freedom and *P* value noted<br>*Give P values as exact values whenever suitable.*
☒	☐	For Bayesian analysis, information on the choice of priors and Markov chain Monte Carlo settings
☒	☐	For hierarchical and complex designs, identification of the appropriate level for tests and full reporting of outcomes
☒	☐	Estimates of effect sizes (e.g. Cohen's *d*, Pearson's *r*), indicating how they were calculated

Our web collection on statistics for biologists contains articles on many of the points above.

Software and code

Policy information about availability of computer code

Data collection	*Provide a description of all commercial, open source and custom code used to collect the data in this study, specifying the version used OR state that no software was used.*
Data analysis	GraphPad Prism7, Flowjo version 10.4, Adobe Photoshop CS4, Ingenuity Pathway Analysis (Qiagen) version 01-04, FastQC, Trimmomatic, HISAT2, featureCounts, DESeq2

For manuscripts utilizing custom algorithms or software that are central to the research but not yet described in published literature, software must be made available to editors/reviewers. We strongly encourage code deposition in a community repository (e.g. GitHub). See the Nature Research guidelines for submitting code & software for further information.

Data

Policy information about availability of data

All manuscripts must include a data availability statement. This statement should provide the following information, where applicable:

- Accession codes, unique identifiers, or web links for publicly available datasets
- A list of figures that have associated raw data
- A description of any restrictions on data availability

There are no restrictions on data availability. RNAseq data has been submitted to the NIH Gene Expression Omnibus (GEO) Repository and GEO accession number is pending

Field-specific reporting

Please select the one below that is the best fit for your research. If you are not sure, read the appropriate sections before making your selection.

☒ Life sciences ☐ Behavioural & social sciences ☐ Ecological, evolutionary & environmental sciences

For a reference copy of the document with all sections, see nature.com/documents/nr-reporting-summary-flat.pdf

Life sciences study design

All studies must disclose on these points even when the disclosure is negative.

Sample size	Sample size was determined based on previous studies. 3 to 10 mice/group/timepoint was sufficient to detect differences between groups with power value 0.8 and a 5% significance level in most of the experiments
Data exclusions	No data were excluded.
Replication	All of data were successfully reproduced.
Randomization	No randomization was done during group allocation. Animals of similar age and sex (liitermates where possible) were used to control for covariates.
Blinding	Investigators were not blinded.

Reporting for specific materials, systems and methods

We require information from authors about some types of materials, experimental systems and methods used in many studies. Here, indicate whether each material, system or method listed is relevant to your study. If you are not sure if a list item applies to your research, read the appropriate section before selecting a response.

Materials & experimental systems

n/a	Involved in the study	
☐	☒	Antibodies
☐	☒	Eukaryotic cell lines
☒	☐	Palaeontology
☐	☒	Animals and other organisms
☐	☒	Human research participants
☒	☐	Clinical data

Methods

n/a	Involved in the study	
☒	☐	ChIP-seq
☐	☒	Flow cytometry
☒	☐	MRI-based neuroimaging

Antibodies

Antibodies used

Antibody (clone), Supplier, Cat. No
HLA-A2 (BB7.2), BioLegend, 343324
CCR7 (G043H7), BioLegend, 353208
CD45RO (UCHL1), BioLegend, 304232
CD8a (SK1), BD Biosciences, 561945
CD3e (SP34-2), BD Biosciences, 557917
CD4 (L200), BD Biosciences, 551980
CD69 (FN50), BioLegend, 310926
CD103 (HML-1), Beckman Coulter, IM1856U
IFNg (B27), BD Biosciences, 554700
TNFa (Mab11), BD Biosciences, 554514
CD11b (M1/70), BD Biosciences, 561114
MHCII I-A/I-E (M5/114.15.2), BioLegend, 107635
CD86 (GL1), BD Biosciences, 563055
CD11c (N418), BioLegend, 117311
CD103 (2E7), eBioscience, 17-1031-80
CD45 (30-F11), BD Biosciences, 563709
CCR7 (4B12), eBioscience, 12-1971-82
NK1.1 (PK136), BioLegend, 108728
CD3 (145-2C11), BD Biosciences, 563004
CD8a (53-6.7), BioLegend, 100743
IFNg (XMG1.2), BD Biosciences, 54411
CD25 (PC61), BD Biosciences, 557192
CD44 (IM7), BioLegend,103059

	CD69 (H1.2F3), BD Biosciences, 562455<br>Granzyme B (GB11), Invitrogen, GRB04
Validation	All the antibodies are from commercial sources and have been validated by the vendors and their validation data are available on the manufacturer's website.

Eukaryotic cell lines

Policy information about cell lines

Cell line source(s)	B16-F10 (ATCC), MC38 (ATCC)
Authentication	Cell line was not authenticated
Mycoplasma contamination	B16-F10 cells tested positive for mycoplasma contamination. All survival and tumor growth experiments were repeated and validated with a certified mycoplasma-free B16-F10 from ATCC. Results of all experiments are reported.<br><br>MC38 tested negative for mycoplasma
Commonly misidentified lines<br>(See ICLAC register)	No commonly misidentified cell lines were used.

Animals and other organisms

Policy information about studies involving animals; ARRIVE guidelines recommended for reporting animal research

Laboratory animals	C57BL/6J (B6) female mice were purchased from The Jackson Laboratory (Bar Harbor, ME) and were maintained in specific-pathogen-free conditions at the University of Minnesota. BRafCA,PtenloxP,Tyr::Cre-ERT2 male and female mice were obtained from the Jackson Laboratory and bred in our animal colony. CD90.1+ OT-I and CD45.1+ OT-I mice were fully backcrossed to C57BL/6J mice and maintained in our animal colony. Sample size was chosen on the basis of previous experience. No sample exclusion criteria were applied. No method of randomization was used during group allocation, and investigators were not blinded. All mice used in experiments were 5-14 weeks of age. All mice were used in accordance with the Institutional Animal Care and Use Committees guidelines at the University of Minnesota.
Wild animals	No wild animals were used
Field-collected samples	No Field-collected samples were used.
Ethics oversight	All mice were used in accordance with the Institutional Animal Care and Use Committees guidelines at the University of Minnesota

Note that full information on the approval of the study protocol must also be provided in the manuscript.

Human research participants

Policy information about studies involving human research participants

Population characteristics	All tumor tissue and blood was obtained from male or female patients age 16-80 undergoing routine surgical resection of solid tumors. Tumor tissue not required for pathological diagnostic procedures was obtained after surgical resection at the University of Minnesota and collected and de-identified by the Tissue Procurement Facility (BioNet, University of Minnesota). Informed consent was obtained from all subjects. The University of Minnesota Institutional Review Board approved all protocols used. Blood was collected in EDTA collection tubes and tumors were collected in RPMI media containing 5% FBS. All samples were stored at 4 degrees until processed (within 24 hours). Specimens reported on were obtained from HLA*A02+ patients that had sufficient tetramer+ cells for analysis by flow cytometry.
Recruitment	Patients undergoing routine surgical tumor resection were informed of the study and consented by M.A.G. (endometrial tumors) or by BioNet staff (all others)
Ethics oversight	Informed consent was obtained from all subjects. The University of Minnesota Institutional Review Board approved all protocols used.

Note that full information on the approval of the study protocol must also be provided in the manuscript.

Flow Cytometry

Plots

Confirm that:

☒ The axis labels state the marker and fluorochrome used (e.g. CD4-FITC).

☒ The axis scales are clearly visible. Include numbers along axes only for bottom left plot of group (a 'group' is an analysis of identical markers).

☒ All plots are contour plots with outliers or pseudocolor plots.

☐ A numerical value for number of cells or percentage (with statistics) is provided.

Methodology

Sample preparation	Mouse: Spleen and lymph nodes were homogenized through a 70 micron filter in RPMI 1640 containing 5% FBS. Single cell suspensions of splenocytes were treated with ACK Lysis buffer to lyse RBC before staining with antibodies. Skin and Braf/Pten tumors were removed and cut into small pieces, followed by treatment with type IV (Sigma, St. Louis, MO, USA) collagenase in 5% RPMI 1640/2 mM MgCl2/2 mM CaCl2 (1h at 37°C, 450 rpm). Skin and tumors were dissociated via gentleMACS dissociator twice and filtered through nytex mesh.<br>Human: Blood was processed by Ficoll gradient. Tumors were minced and digested in Collagenase type IV (endometrial) or Collagenase Type I (all others). They were then dissociated via gentleMACS Dissociator once (glioblastoma or brain metastases) or twice (all others) and lymphocytes purified on a 44/67% Percoll (GE Healthcare) gradient.
Instrument	BD LSR II and LSR fortessa
Software	BD FACS DIVA was used for collection and analysis was done in FlowJo 10.4
Cell population abundance	Not applicable
Gating strategy	Starting cells were gated by FSC/SSC gates and then with viability dye (GhostDye) to select live population. For human samples, these cells were further gated by CD8a+/CD4- then CD3+, and tetramer marker was gated on to focus on virus-specific CD8+ T cells. Expression of CD69, CD103, CCR7, IFNg and TNFa was examined on these populations as indicated in the figure legends. For mouse samples, after FSC/SSC and gating on live cells, lymphocytes were further gated by CD8a+ and gating on congenic marker (CD90.1 or CD45.1) was done to examine virus-specific CD8+ T cells, or gating of congenic marker-negative, CD44hi cells was done to examine non-peptide specific memory T cells. Expression of IFNg, CD25 and granzyme B was examined. NK cells were gated on CD8-/CD4-/NK1.1+ after live gate and expression of granzyme B was examined. DCs were gated on MHCIIhi/ CD11b-/CD11c+/CD103+ after live gate and expression of CD86 and CCR7 was examined.

☒ Tick this box to confirm that a figure exemplifying the gating strategy is provided in the Supplementary Information.
